# Mesenchymal stem cells protect against sepsis-associated acute kidney injury by inducing Gal-9/Tim-3 to remodel immune homeostasis

**DOI:** 10.1080/0886022X.2023.2187229

**Published:** 2023-03-08

**Authors:** Congjuan Luo, Feng Luo, Lin Che, Hui Zhang, Long Zhao, Wei Zhang, Xiaofei Man, Quandong Bu, Hong Luan, Bin Zhou, Haiyan Zhou, Yan Xu

**Affiliations:** Department of Nephrology, The Affiliated Hospital of Qingdao University, Qingdao, Shandong, People's Republic of China

**Keywords:** Mesenchymal stem cells, sepsis-associated acute kidney injury, Gal-9/Tim-3, immune homeostasis

## Abstract

**Objective:**

The present study investigated the specific mechanism by which mesenchymal stem cells (MSCs) protect against sepsis-associated acute kidney injury (SA-AKI).

**Methods:**

Male C57BL/6 mice underwent cecal ligation and puncture surgery to induce sepsis and then received either normal IgG or MSCs (1 × 10^6^ cells, intravenously) plus Gal-9 or soluble Tim-3 3 h after surgery.

**Results:**

After cecal ligation and puncture surgery, the mice injected with Gal-9 or MSCs plus Gal-9 had a higher survival rate than the mice in the IgG treatment group. Treatment with MSCs plus Gal-9 decreased serum creatinine and blood urea nitrogen levels, improved tubular function recovery, reduced IL-17 and RORγt levels and induced IL-10 and FOXP3 expression. Additionally, the Th17/Treg cell balance was altered. However, when soluble Tim-3 was used to block the Gal-9/Tim-3 pathway, the septic mice developed kidney injury and exhibited increased mortality. Treatment with MSCs plus soluble Tim-3 blunted the therapeutic effect of MSCs, inhibited the induction of Tregs, and suppressed the inhibition of differentiation into Th17 cells.

**Conclusion:**

Treatment with MSCs significantly reversed the Th1/Th2 balance. Thus, the Gal-9/Tim-3 pathway may be an important mechanism of MSC-mediated protection against SA-AKI.

## Introduction

Acute kidney injury (AKI) is a common critical illness involving multiple systems. AKI is a syndrome associated with the rapid deterioration of renal function caused by various etiologies. Sepsis is one of the most important causes of AKI, and up to 60% of patients with sepsis have AKI [1]. Sepsis-associated AKI (SA-AKI) substantially increases the difficulty of sepsis treatment and medical costs. In recent years, despite the rapid development of critical care medicine and nephrology, the mortality rate for SA-AKI has remained high. Therefore, the prevention and treatment of SA-AKI is important. Stem cell research is currently a hotspot in the treatment of AKI. As a type of adult stem cell, mesenchymal stem cells (MSCs) not only show proliferation and differentiation but also immune regulatory functions [2]. Although MSCs have been studied in relation to SA-AKI [3], their specific protective mechanisms in SA-AKI remain unclear.

Galectin-9 (Gal-9) belongs to the galectin family and is a natural ligand of Tim-3. Almost all cells express Gal-9 [4]. However, in the inactivated immune system, Gal-9 is mainly expressed by naive CD4^+^ effector T cells and regulatory T (Treg) cells. After activation, Gal-9 on the surface of effector T cells is downregulated, but Gal-9 on the surface of Treg cells is maintained [5]. Studies have confirmed that the Gal-9/Tim-3 pathway can negatively regulate the Th1 immune response, and the binding of Gal-9 and Tim-3 can induce the apoptosis of Th1 and Th17 cells that express Tim-3, thereby inducing Treg cells and immune tolerance. The Gal-9/Tim-3 pathway may have evolved to control the population expansion and tolerance of Th1 cells in the immune compartment and to prevent prolonged inflammation in target tissues [6]. Studies have reported that the Gal-9/Tim-3 pathway is involved in immune disorders in SA-AKI [7].

In recent years, the protective effects of MSCs in sepsis and AKI have been confirmed. For example, MSCs could significantly improve the survival rate of septic mice and related organ damage [3]. The underlying mechanism includes the secretion of prostaglandin E2 to induce the conversion of M1 macrophages to M2 macrophages [8]. MSC intervention significantly increased the percentage of peripheral blood Treg cells and improved the function of Treg cells, restoring immune homeostasis in septic rats [9].

MSCs can improve AKI caused by ischemia/reperfusion injury (IRI), crush syndrome, and sepsis, and in determining the potential mechanisms, researchers have mainly focused on the following factors: (1) paracrine and endocrine mechanisms – MSCs secrete trophic growth factors or inflammatory regulatory factors, inhibit the apoptosis of vascular endothelial cells and tubular epithelial cells, promote local cell regeneration, and reduce local inflammatory cell infiltration in the kidneys [10]; and (2) immunomodulatory function – MSCs are involved in the induction of Tregs and the conversion of M1 macrophages to M2 macrophages [8]. Recent studies have also found that MSCs can remodel the Th1/Th2 balance, thereby reducing the production of interferon γ (IFN-γ) by CD4+ Th1 cells, reducing the release of interleukin-17 (IL-17) by Th17 cells, and increasing the release of IL-4 by Th2 cells [11]. More importantly, MSCs can promote the production of IL-10 by Treg cells. *In vitro*, when cocultured with MSCs and naive T cells, MSCs can significantly increase the differentiation of naive T cells into Treg cells [12]. Recent studies have found that MSCs can inhibit T cell function by secreting Gal-1 and Gal-3, and silencing the expression of Gal-1 and/or Gal-3 in MSCs with small interfering RNA nearly abolished the immunoregulatory potential of MSCs [13]. Our previous studies confirmed that MSCs can alleviate SA-AKI [3]. However, the mechanism by which MSCs alleviates SA-AKI remains unclear. In this study, we blocked or activated the Gal-9/Tim-3 pathway to reveal the specific mechanism by which MSCs protect against SA-AKI.

## Methods

### Animal protocols

Male C57BL/6 mice (6–8 weeks old) were provided by the Experimental Animal Center of Qingdao University. The experiments performed were approved by the Experimental Animal Welfare Ethics Committee of Qingdao University (No. 202105C5742202106034); all operations performed in the animal experiments were in compliance with the ethical standards of the Chinese Association of Laboratory Animal Care.

### Mouse model of cecal ligation and puncture (CLP)

The sepsis model of CLP was generated as described previously [3]. Mice were anesthetized with 2% pentobarbital, the abdominal cavity was opened layer by layer to locate the cecum, and the distal end was carefully dissociated. The feces in the cecum were gently squeezed toward the distal end of the cecum. Seventy-five percent of the cecum was ligated with a 4-0 silk suture, and the cecum was punctured twice with a 21-gauge needle. A small amount of feces was squeezed on the surface of the cecum, and then, the cecum was retracted into the abdominal cavity and sutured. In the sham operation group, only cecal separation was performed, without ligation and puncture.

### MSC culture

Bone marrow-derived MSCs from C57/BL6 mice were purchased from Cyagen Biosciences (Sunnyvale, Calif). The culture process was conducted according to the manufacturer’s instructions. Sixth- to eighth-generation MSCs were collected for use. At 3 h after CLP, the mice were injected with 10^6^ MSCs, IgG (BD Biosciences, San Diego, CA), Gal-9 (100 µg/mouse, R&D Systems, Minneapolis, MN), or soluble Tim-3 (100 µg/mouse, eBioscience, San Diego, CA) *via* the tail vein.

### Survival analysis

Mice in each group (20 per group) were observed every 24 h for 7 d. The mice that survived were sacrificed under diethyl ether anesthesia after 7 d.

### Assessment of kidney function

The mice were sacrificed under diethyl ether anesthesia at 24 h (*n* = 8 per group) after surgery. Blood (500 μL/mouse) was collected from the tail vein and then centrifuged at 3000 rpm for 15 min; the upper serum layer was retained for the determination of serum creatinine (Cr) and blood urea nitrogen (BUN) levels.

### Histopathological analysis

Kidneys were harvested 24 h after CLP. The kidneys were collected and placed in 10% formaldehyde. After fixation at 4 °C for 48 h, the tissue was dehydrated and embedded in paraffin for pathological staining. Pathological scoring was performed using a double-blind method. Tubular injuries were scored by three independent observers. Renal tissue lesions, including the shedding of the brush border, tubular necrosis, exfoliation of the epithelial cells, cast deposition, and vascular congestion, were scored as 0–5 according to the intensity of the damage. Semiquantitative scoring was performed based on previously reported methods [14]: 0 = no necrosis, 1 ≤ 10%, 2 ≤ 11–25%, 3 = 26–45%, and 4 = 46–75%. Six mice in each group were observed, and at least 20 high-magnification fields (magnification: 400×) were visualized in each section.

### Real-time PCR

Total RNA was extracted from renal sample homogenates using TRIzol reagent (Invitrogen, Carlsbad, CA, USA). Reverse transcription into complementary DNA (cDNA) was performed using a TaqMan reverse transcription kit (Applied Biosystems, Foster City, CA, USA). Real-time PCR was performed using SYBR Green Master Mix (Toyobo) and an ABI 7500 real-time PCR system (Applied Biosystems, Foster City, CA). GAPDH levels were used for normalization. The sequences of the real-time PCR primers are shown in Table 1.

**Table 1.** Primers used for real-time PCR.

Gene	Forward primers(5′–3′)	Reverse primer(5′–3′)
IL-17	TGGACTCTCCACCGCAATG	TGGGGGTTTCTTAGGGGTCA
IL-10	AGGCAGCCTTGCAGAAAAGA	GCTCCACTGCCTTGCTCTTA
RORγt	CCC ATC TATGAG GGTTACGC	TTT AAT GTC ACG CACGAT TTC
FOXP3	ACCGTA TCT CCT GAGTTC CAT	GTCCAGCTTGACCACAGTTTAT
GAPDH	CCGATGCCTTTCATCACCACC	CACCTTGAGGCAGTGAGCTTC

### Western blotting

The RORγt, IL-17, Foxp3, and IL-10 protein levels were measured *via* western blotting (*n* = 5 per group). Total proteins were extracted from the kidney tissue using a complete radioimmunoprecipitation assay (RIPA) lysis buffer, and protein levels were measured using a bicinchoninic acid assay kit (Thermo Scientific, Bremen, Germany). A total of 100 μg of protein was separated and transferred onto polyvinylidene difluoride (PVDF) membranes. Then, the primary antibodies anti-RORγt, anti-IL-17, anti-Foxp3, anti-IL-10 (Santa Cruz Biotechnology, Inc., Santa Cruz, CA) and anti-actin (Cell Signaling Technology) were incubated with the membranes at 4 °C overnight, and horseradish peroxidase-conjugated anti-rabbit/mouse IgG (Santa Cruz Biotechnology) was used as the secondary antibody. Protein levels were semiquantitatively determined based on the optical density using ImageJ software and are expressed as the protein/actin ratio. Sample quantification was performed twice.

### Flow cytometry

Antibodies targeting the following proteins were used for multicolor flow cytometric analysis: CD4-PE-CY7 (FITC, GK1.5, eBioscience), CD25-APC (BioLegend), Foxp3-PC5 (FJK-16S; eBioscience), and IL-17-PE (eBioscience). For staining of intracellular markers, cells were incubated for 20 min at 4 °C in Cytofix/Cytoperm (Biolegend) to permeabilize cell membranes. Intracellular markers were stained according to standard laboratory procedures. All data were acquired using a FACSCalibur flow cytometer (BD Biosciences) and analyzed with FlowJo software (Tree Star, Ashland, OR, USA).

### Statistical methods

The survival rate of mice in each group was evaluated using Kaplan–Meier analysis. The results are presented as the mean ± SD. Groups were compared using one-way ANOVA or a Kruskal–Wallis *H* test, followed by Tukey’s or Dunn’s test. mRNA levels were calculated using the 2^−ΔΔCT^ method. *p* < .05 was considered statistically significant. All statistical analyses were performed using SPSS statistical software (SPSS version 10.1).

## Results

1. Effect of exogenous Gal-9 activation of Gal-9/Tim-3 on 7-d survival in septic mice treated with MSCs.

**Figure 1.**
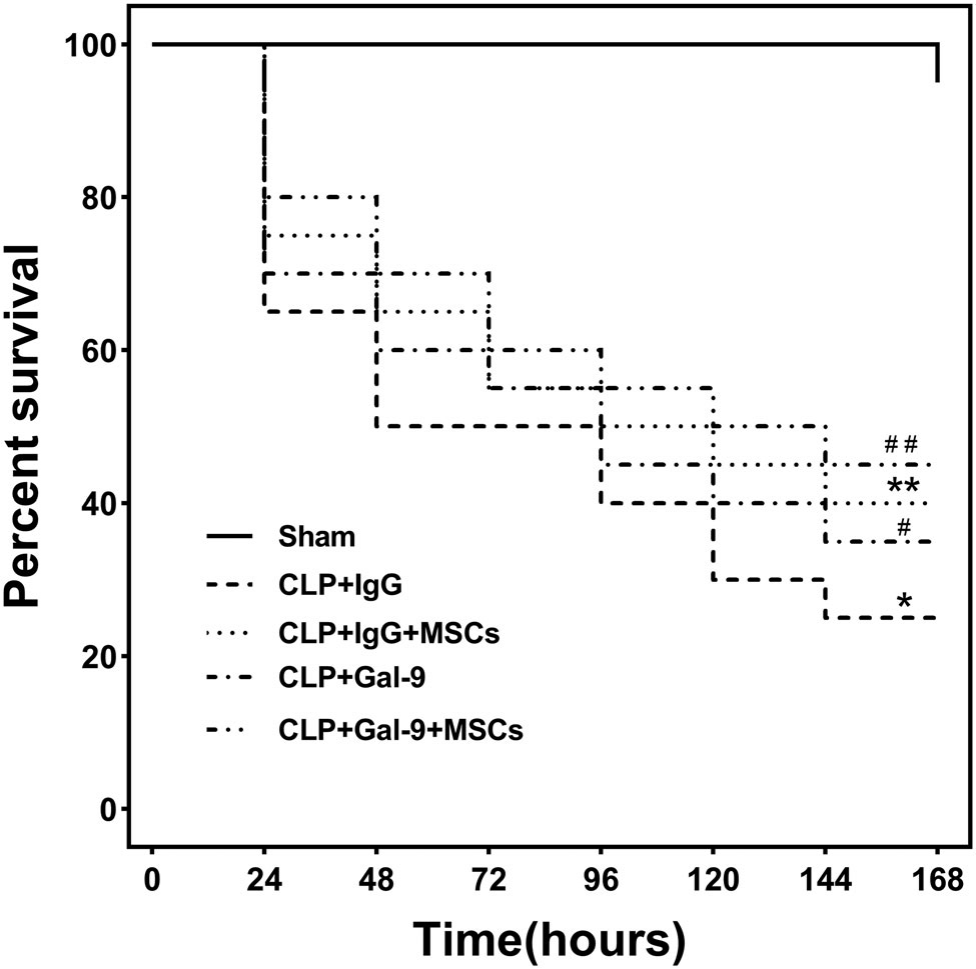
Seven-day survival rate for mice in each group: Each group consisted of 20 animals. Kaplan-Meier curves represent the survival rate in each group. compared with CLP + IgG group, ^#^*p* < .05, **p < .05; compared with the CLP + Gal-9 group, ^##^*p* > .05.

In the absence of antibiotics, we established a sepsis model *via* CLP surgery. High mortality rates were observed at 24 h after CLP surgery, indicating that the sepsis model was successfully established. On day 7, the survival rate for the mice in the CLP + IgG group was only 25%, while that for mice in the CLP + Gal-9 + MSC group was 45%. Compared with those in the CLP + IgG group, the 7-d survival rates of the mice in the CLP + IgG + MSC, CLP + Gal-9, and CLP + Gal-9 + MSC groups were significantly higher; however, the difference between the CLP + Gal-9 group and CLP + Gal-9 + MSC group was not significant (Figure 1).

1. Effect of exogenous Gal-9 activation of Gal-9/Tim-3 on renal function in septic mice treated with MSCs.

**Figure 2.**
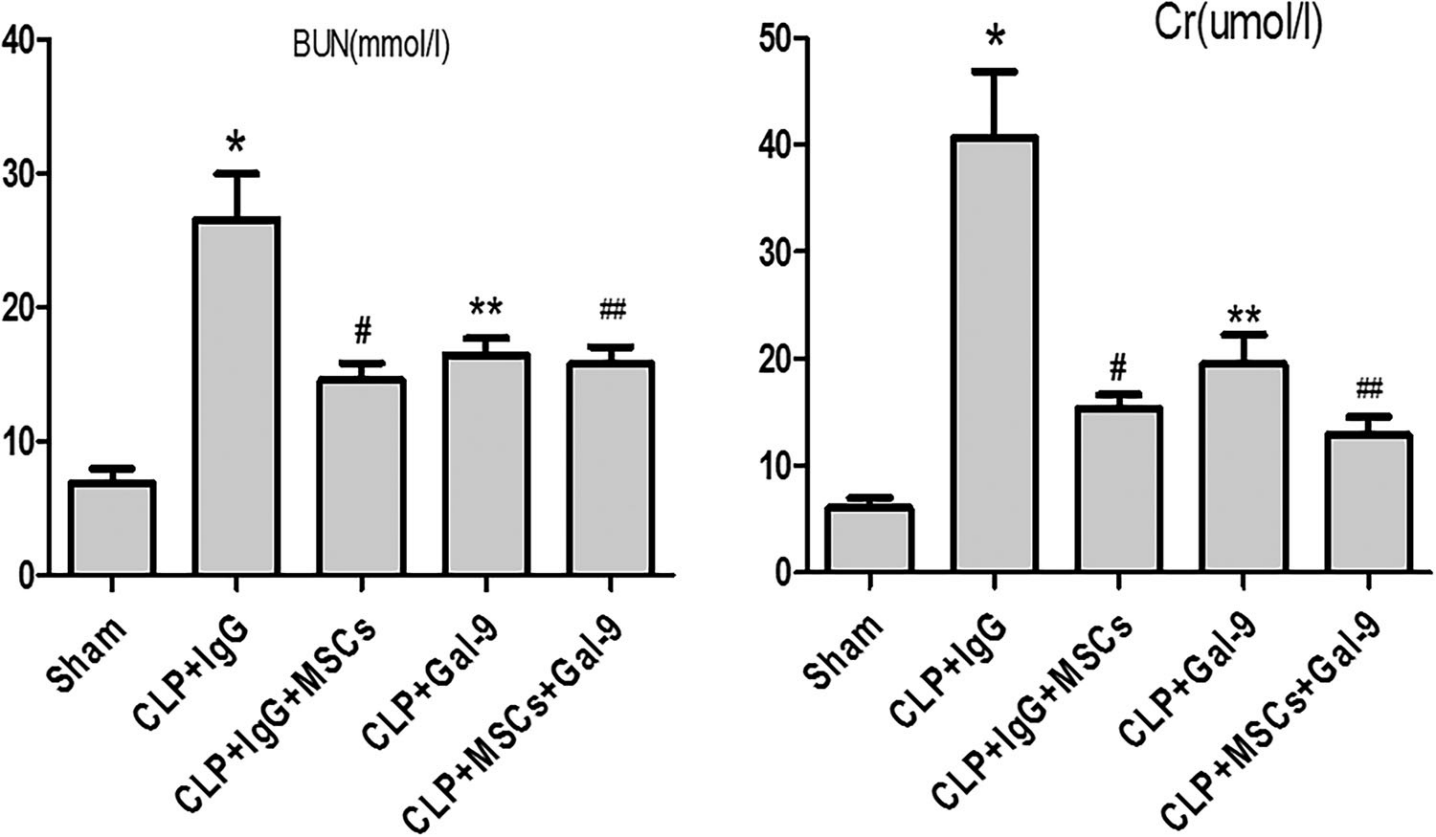
The levels of serum Cr and BUN 24 h after CLP injury in each group: values are mean ± SD; compared with CLP + IgG group, ^#^*p* < .05, **p < .05; compared with the CLP + Gal-9 group, ^##^*p* > .05.

The Cr and BUN levels were significantly increased in the CLP + IgG, CLP + IgG + MSC, CLP + Gal-9, and CLP + Gal-9 + MSC groups, suggesting that the model was successfully established. Compared with those in the CLP + IgG group, the Cr and BUN levels were significantly decreased in the CLP + IgG + MSC and CLP + Gal-9 groups. Although the Cr and BUN levels in the CLP + Gal-9 + MSC group were decreased compared with those in the CLP + Gal-9 group, the difference was not significant (Figure 2).

1. Effect of exogenous Gal-9 activation of Gal-9/Tim-3 on kidney pathology in septic mice treated with MSCs.

**Figure 3.**
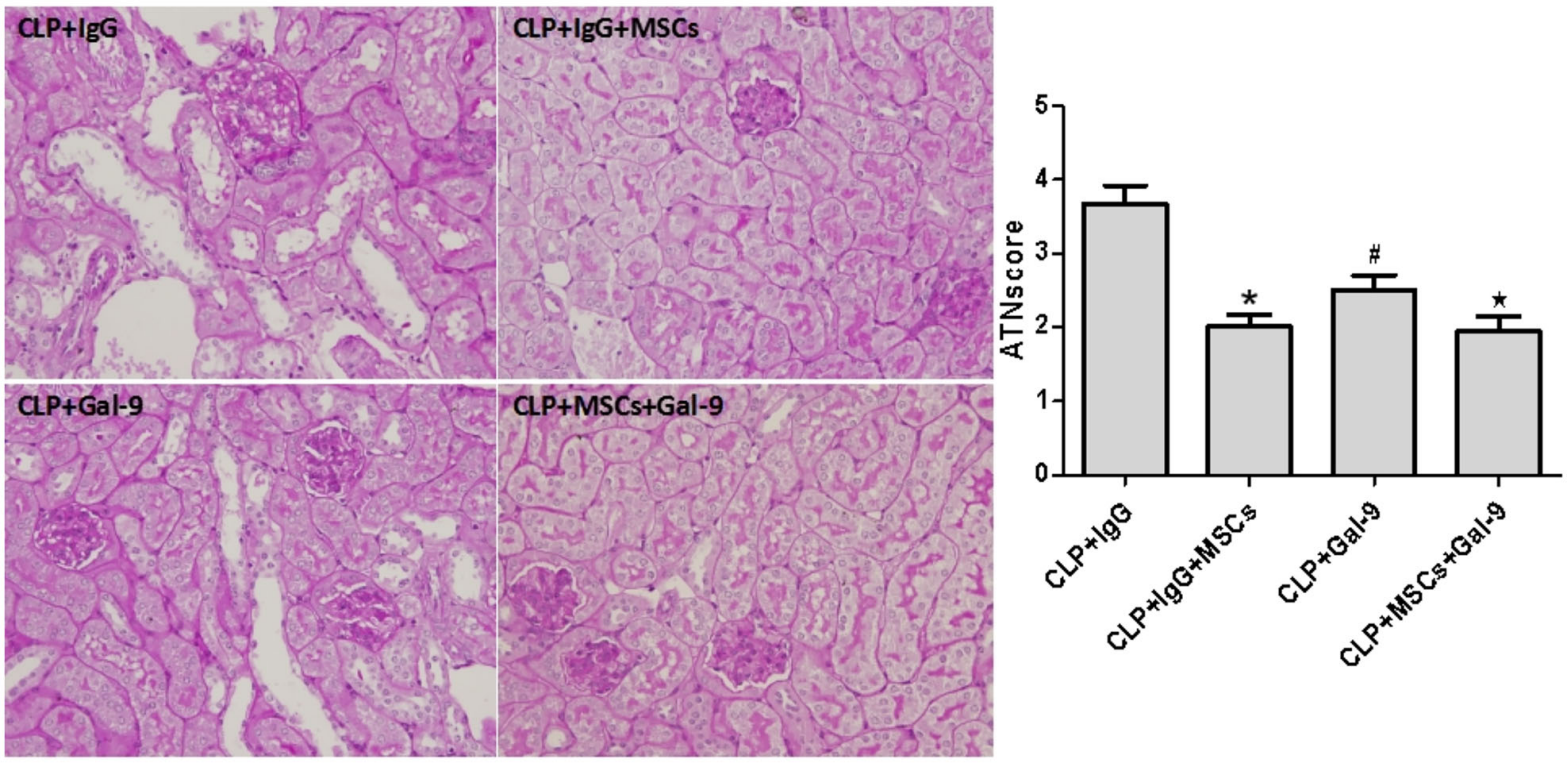
The acute tubular necrosis (ATN) scores 24 h after CLP in each group: values are mean ± SD; compared with CLP + IgG group, *p < .05,^#^*p* < .05; compared with the CLP + Gal-9 group, ^★^*p* > .05.

As described previously, the pathological changes in the kidneys in all septic groups manifested as brush border loss, tubular degeneration, and exfoliation of the epithelial cells of both the proximal and the thick ascending limb. Compared with those of the CLP + IgG group, the tubular injury scores of the CLP + IgG + MSC and CLP + Gal-9 groups were significantly lower. Although the tubular injury scores of the CLP + Gal-9 + MSC group were lower than those of the CLP + Gal-9 group, the difference was not significant (Figure 3).

1. Effect of exogenous Gal-9 activation of Gal-9/Tim-3 on Treg and Th17 cells in the kidneys of septic mice treated with MSCs.

The percentage of Th17 cells and the mRNA (Figure 4(A)) and protein expression (Figure 4(B)) levels of RORγt and IL-17 were higher, while the FoxP3 and IL-10 mRNA and protein expression levels were lower in the CLP + IgG group. These findings are consistent with previous studies, indicating that Tregs and Th17 cells play key roles in the pathogenesis of sepsis. The percentage of Th17 cells was significantly lower in the CLP + IgG + MSC intervention group than in the CLP + IgG group (Figure 4(C)), while the percentage of Treg cells was significantly higher (Figure 4(D)). This finding suggests that treatment with MSCs can reduce the Th17/Treg ratio in SA-AKI. We speculate that MSCs may alter the Th17/Treg cell balance *via* Gal-9/Tim-3 to protect against SA-AKI. To verify this hypothesis, we used exogenous Gal-9 to activate the Gal-9/Tim-3 pathway. We found that the percentage of Th17 cells in the kidney was significantly decreased (Figure 4(C)), the RORγt and IL-17 mRNA and protein expression levels were significantly reduced (Figure 4(A,B)), and the FoxP3 and IL-10 mRNA and protein expression levels were increased (Figure 4(A,B)). However, no significant differences between the CLP + Gal-9 group and the CLP + Gal-9 + MSC group were found. This finding demonstrates that the Gal-9/Tim-3 pathway is very important in the regulation of the Th17/Treg ratio in MSCs.

Figure 4.(A) The mRNA expression of IL-17, RORγt, FoxP3 and IL-10 in kidney tissues 24 h after CLP operation. compared with CLP + IgG group, *p < .05, ^#^*p* < .05; compared with the CLP + Gal-9 group, ^★^*p* > .05. (B) The Western blotting expression and quantitative analysis of IL-17, RORγt, FoxP3 and IL-10 in kidney tissues. The protein levels are presented as mean ± SD. compared with CLP + IgG group, *p < .05, ^#^*p* < .05; compared with the CLP + Gal-9 group, ^★^*p* > .05. (C) The percentage of Th17 cells relative to CD4+ T cells were measured by flow cytometry after the preparation of single-cell suspensions. the values are mean ± SD. Comparisons between groups were performed using Student *t*-test. compared with CLP + IgG group, *p < .05, ^#^*p* < .05; compared with the CLP + Gal-9 group, ^★^*p* > .05. (D) The percentage of Treg cells relative to CD4+ T cells were measured by flow cytometry after the preparation of single-cell suspensions. the values are mean ± SD. Comparisons between groups were performed using Student t-test. compared with CLP + IgG group, *p < .05, ^#^*p* < .05; compared with the CLP + Gal-9 group, ^★^*p* > .05.

**Figure:**
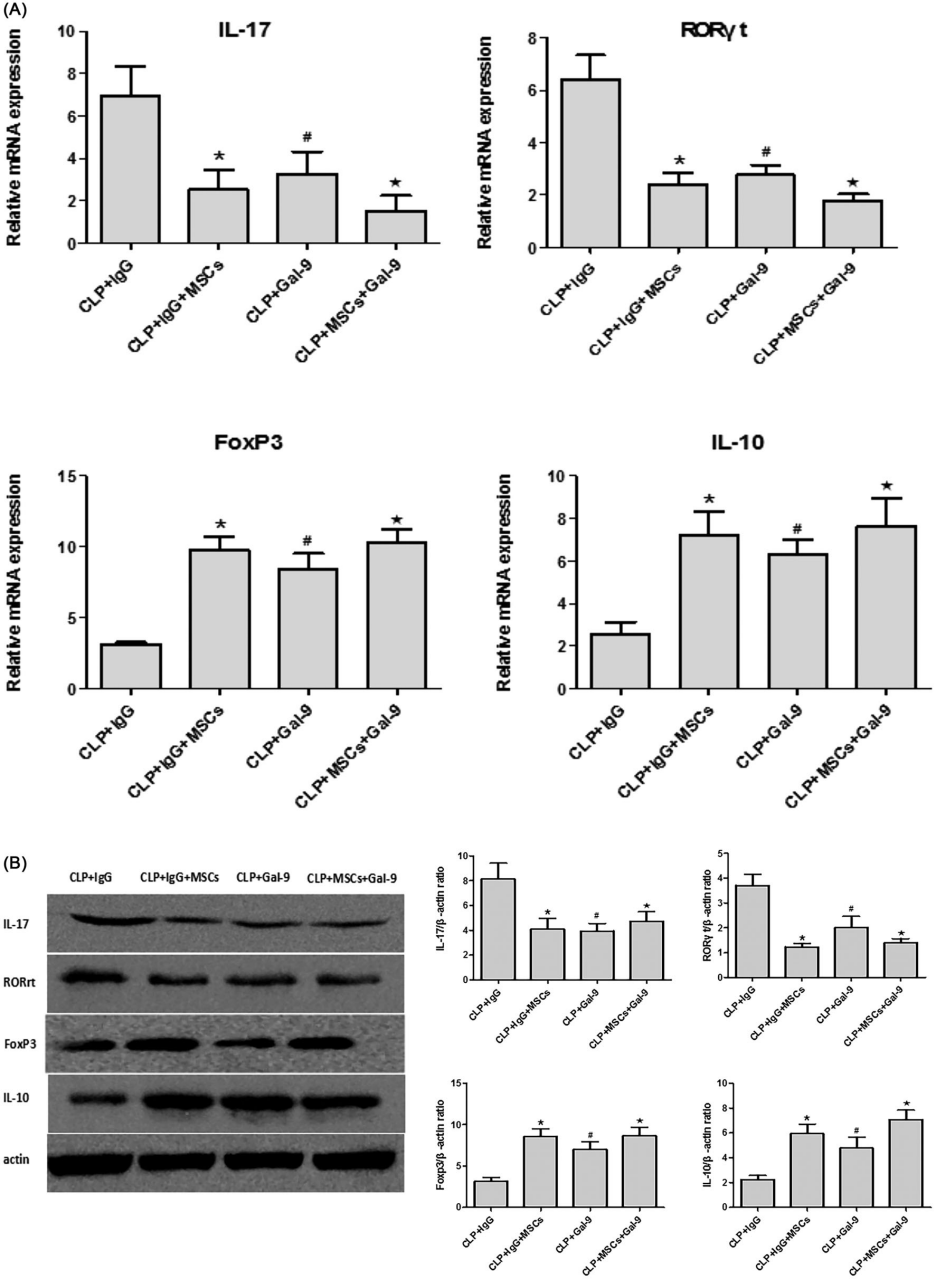

**Figure:**
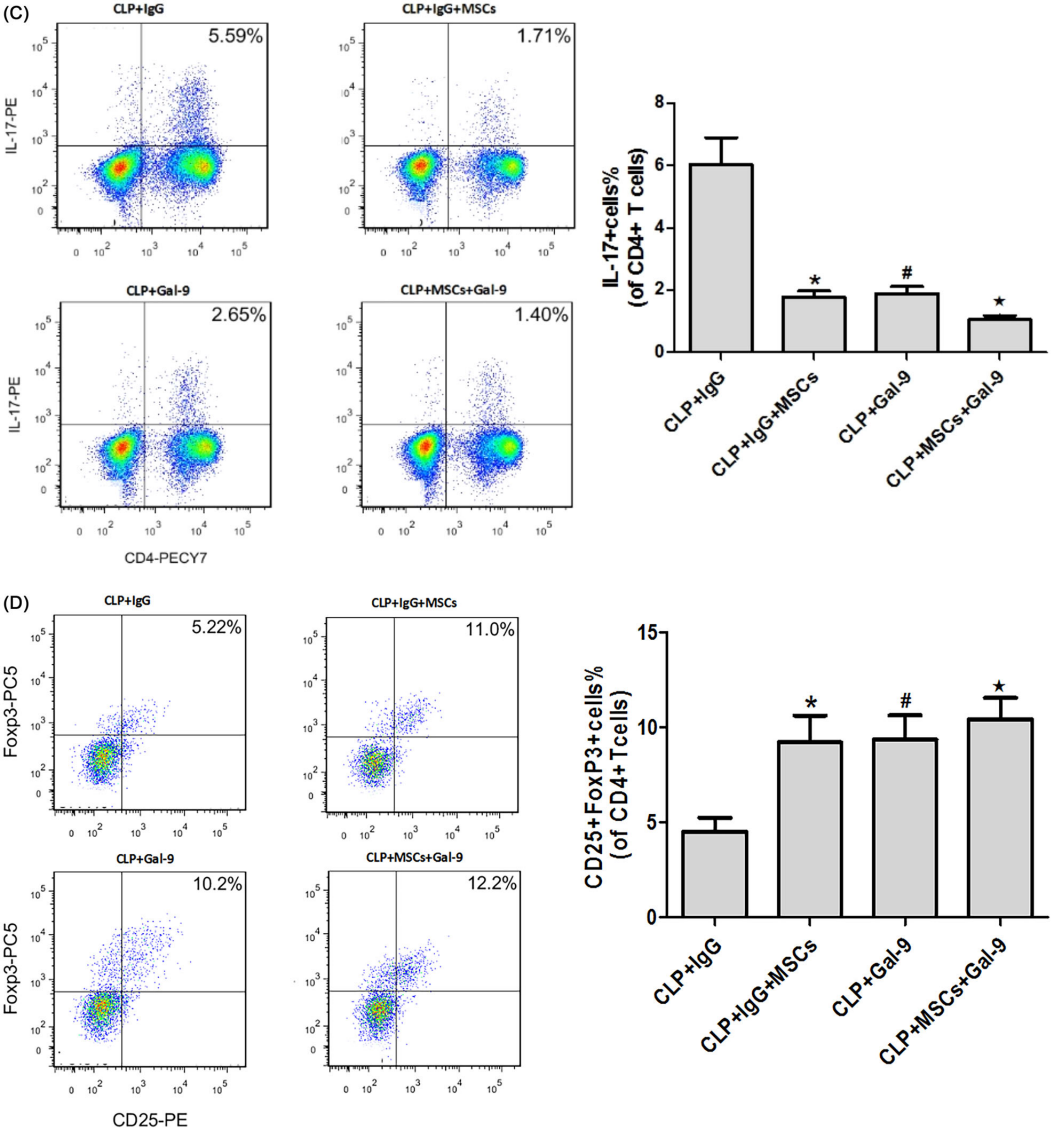

1. Effect of blocking Gal-9/Tim-3 on the 7-day survival of septic mice treated with MSCs.

**Figure 5.**
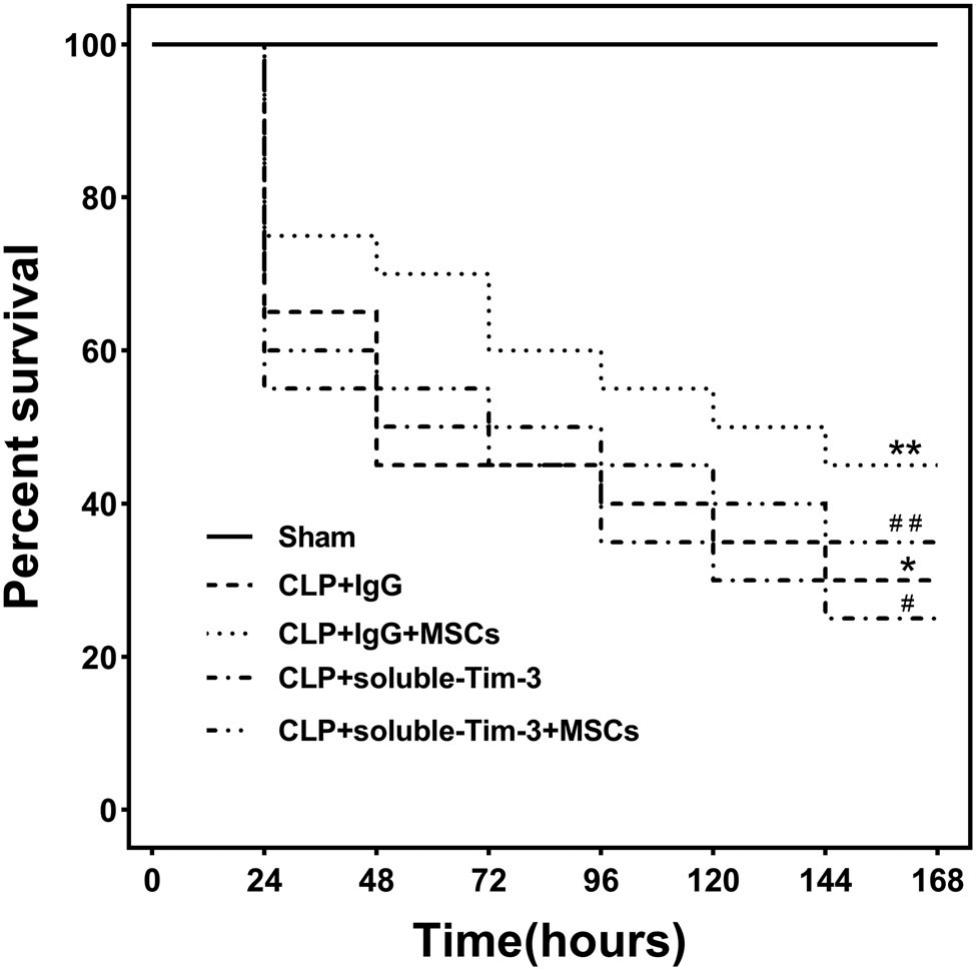
Seven-day survival rate for mice in each group: Each group consisted of 20 animals. Kaplan-Meier curves represent the survival rate in each group. compared with the CLP + IgG group, ^#^*p* < .05, **p > .05; compared with CLP + soluble-Tim-3 group, ^##^*p* > .05.

We used soluble Tim-3 to block the Gal-9/Tim-3 pathway *in vivo*. On day 7, no significant difference was observed between the CLP + soluble Tim-3 group and the CLP + soluble Tim-3 + MSC group (Figure 5).

1. Effect of blocking Gal-9/Tim-3 on renal function in septic mice treated with MSCs.

**Figure 6.**
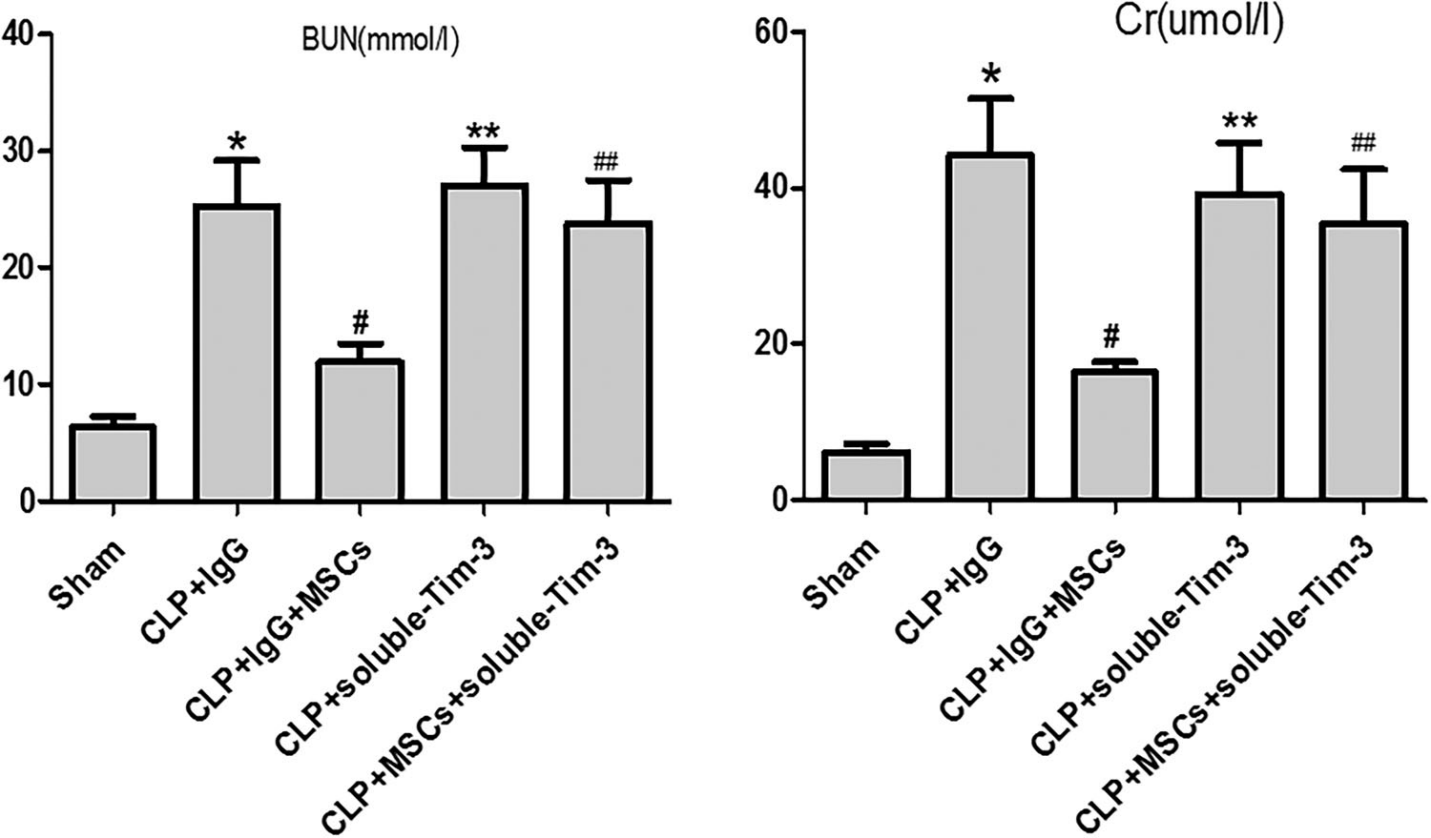
The levels of serum Cr and BUN 24 h after CLP injury in each group: values are mean ± SD; *n* = 8 in each group. compared with the CLP + IgG group, ^#^*p* < .05, **p > .05; compared with CLP + soluble-Tim-3 group, ^##^*p* > .05.

Compared with those in the CLP + IgG group, the Cr and BUN levels in the CLP + IgG + MSC group were significantly lower. However, the Cr and BUN levels in the CLP + soluble Tim-3 group were increased. Although the Cr and BUN levels in the CLP + soluble-Tim-3 + MSC group were lower than those in the CLP + soluble-Tim-3 group, the difference between the groups was not significant (Figure 6).

1. Effect of blocking Gal-9/Tim-3 on pathological damage in the kidneys of septic mice treated with MSCs.

**Figure 7.**
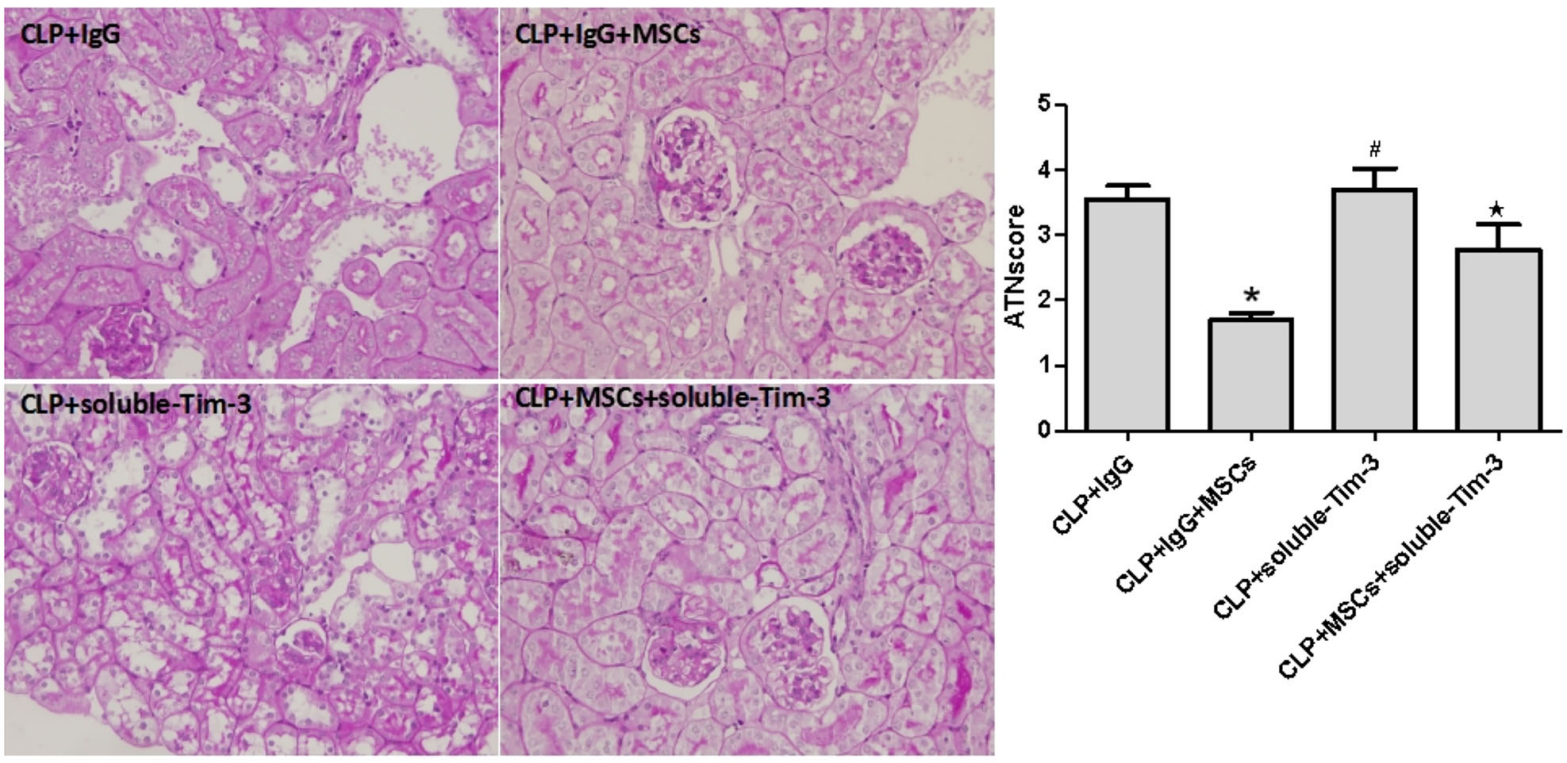
The acute tubular necrosis (ATN) scores 24 h after CLP in each group: values are mean ± SD; *n* = 8 in each group. compared with the CLP + IgG group, *p < .05, ^#^*p* > .05; compared with CLP + soluble-Tim-3 group, ^★^*p* > .05.

The renal tubular injury score in the CLP + IgG + MSC group was significantly lower than that in the CLP + IgG group. However, the renal tubular injury score in the CLP + soluble Tim-3 group did not decrease significantly, indicating that soluble Tim-3 did not protect against SA-AKI. Compared with that of the CLP + soluble-Tim-3 group, the renal tubular injury score in the CLP + soluble-Tim-3 + MSC group did not significantly decrease (Figure 7). These results indicate that blocking Gal-9/Tim-3 disrupted the protective effect of MSCs in SA-AKI.

1. Effect of blocking Gal-9/Tim-3 on Treg and Th17 cells in the kidneys of septic mice treated with MSCs.

We blocked the Gal-9/Tim-3 pathway with soluble Tim-3. Compared with that in the CLP + IgG group, the percentage of TH17 cells in the kidney tissue was significantly decreased in the CLP + IgG + MSC group, and the RORγt and IL-17 mRNA (Figure 8(A)) and protein (Figure 8(B)) expression levels were significantly decreased. In addition, the percentage of Treg cells in the kidney tissue of the CLP + IgG + MSC group was significantly increased, and the FoxP3 and IL-10 mRNA (Figure 8(A)) and protein (Figure 8(B)) levels were significantly different from those in the CLP + IgG group. Through blocking of the Gal-9/Tim-3 pathway with soluble Tim-3, the regulation of the Th17/Treg (Figure 8(C,D)) ratio by MSCs was no longer significant, indicating that regulation of the Th17/Treg ratio *via* the Gal-9/Tim-3 pathway plays an important role in the MSC-induced protection against SA-AKI.

Figure 8.(A) The mRNA expression of IL-17, RORγt, FoxP3 and IL-10 in kidney tissues 24 h after CLP operation. compared with the CLP + IgG group, *p < .05, ^#^*p* > .05; compared with CLP + soluble-Tim-3 group, ^★^*p* > .05. (B) The Western blotting expression and quantitative analysis of IL-17, RORγt, FoxP3 and IL-10 in kidney tissues. The protein levels are presented as mean ± SD. compared with the CLP + IgG group, *p < .05, ^#^*p* > .05; compared with CLP + soluble-Tim-3 group, ^★^*p* > .05. (C) The percentage of Th17 cells relative to CD4+ T cells were measured by flow cytometry after the preparation of single-cell suspensions. the values are mean ± SD. Comparisons between groups were performed using Student *t*-test. compared with the CLP + IgG group, *p < .05, ^#^*p* > .05; compared with CLP + soluble-Tim-3 group, ^★^*p* > .05. (D) The percentage of Treg cells relative to CD4+ T cells were measured by flow cytometry after the preparation of single-cell suspensions. the values are mean ± SD. Comparisons between groups were performed using Student *t*-test. compared with the CLP + IgG group, *p < .05, ^#^*p* > .05; compared with CLP + soluble-Tim-3 group, ^★^*p* > .05.

**Figure:**
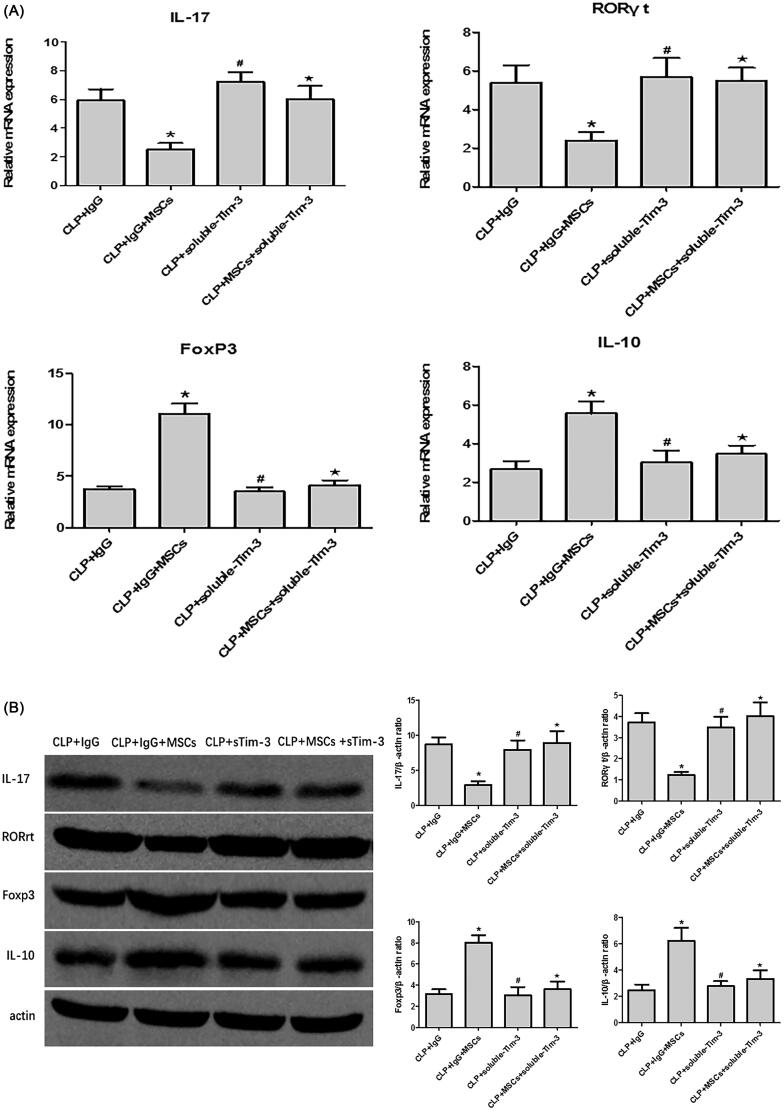

**Figure:**
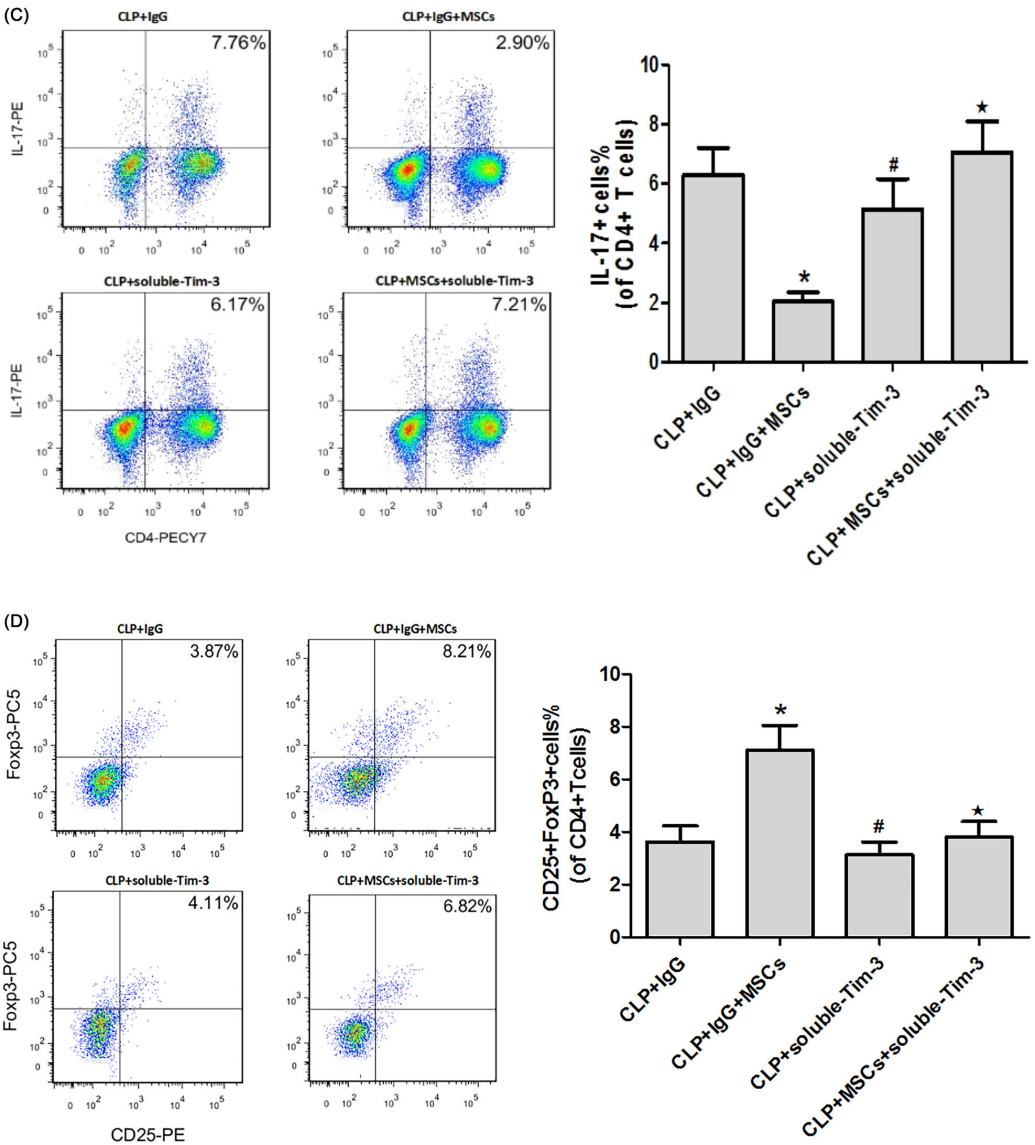

## Discussion

Sepsis is a life-threatening condition caused by a microbial infection that triggers an excessive inflammatory response. The results of the present study confirmed the poor mortality after the CLP operation, indicating the development of sepsis. In addition to the direct damage caused by pathogens and their toxins, the immune function also plays a crucial role in the septic process. Kinsey et al. [15] used antibodies (PC61) to eliminate Treg cells in mice 5 d before constructing an IRI-induced AKI model. As a result, the infiltration of inflammatory cells in the kidney was significantly increased, and the secretion of IL-6, TNF-α, and TGF-β (but not IL-10) was increased, thereby exacerbating the renal injury. To further verify the relationship between Treg cells and AKI, Kinsey et al. injected Treg cells from wild-type mice and Foxp3-deficient mice into RAG-1 mice (lacking T and B cells) before AKI modeling. The former alleviated AKI, while the latter increased the infiltration of neutrophils and macrophages in the kidneys, thereby aggravating AKI. These findings demonstrate that Tregs can directly inhibit innate immune responses in IRI-AKI.

Lee et al. [16] used PC61 to eliminate Treg cells in mice 24 h after AKI injury and found that AKI still led to increased Cr, while the transfer of Treg cells from wild-type mice 24 h after injury significantly reduced tubular necrosis scores and promoted tubular epithelial cell regeneration, suggesting that Treg cells are involved in AKI repair. In our study, although the local Treg cells in septic kidney tissues were slightly increased, the imbalance of Th1/Th2 to Th1 cells was confirmed to eventually result in kidney injury.

As the main source of IL-17, Th17 cells can recruit neutrophils and promote inflammatory mediator release. Studies have shown that IL-17 increases in a time-dependent manner in the peripheral blood of septic mice [17]. The injection of antibodies to neutralize IL-17 before CLP injury significantly improved survival and reduced the expression of inflammatory factors and chemokines. Even delaying the injection of anti-IL-17 antibody 12 h after CLP injury still resulted in an improvement in sepsis [17,18]. In addition to septic animal models, Brunialti et al. [19] detected significantly increased IL-17 and an increased percentage of IL-17-producing Th17 cells in the peripheral blood of patients with sepsis. In recent years, the roles of IL-17 and Th17 cells in kidney diseases have been established. Elevated IL-17 expression has been detected in kidney tissues from patients with focal segmental glomerulosclerosis (FSGS), membranoproliferative glomerulonephritis, and minimal change disease [20]. Li et al. [21] found that IL-17 was significantly increased in an IRI-AKI mouse model. These researchers reported that IL-17a^−/−^ mice or mice with IL-17A neutralization had significantly decreased neutrophil infiltration and tissue injury 24 h after kidney IRI, indicating that IL-17 participates in the innate immune inflammation associated with kidney IRI. Our study identified a significant increase in Th17 cells in the kidneys of septic mice. Although the percentage of Treg cells also increased slightly, this increase was much lower than the increase in Th17 cells, suggesting a role of the Th17-mediated Th1 inflammatory response in the pathogenesis of SA-AKI.

Recent studies have reported that in addition to the regulation of Th1 cells, the Gal-9/Tim-3 signaling pathway is also involved in the regulation of Th17 and Treg cells [22,23]. When the Tim-3 pathway was decreased and/or blocked in sepsis by an antagonist monoclonal antibody against Tim-3 and/or soluble Tim-3 protein, the levels of Th1- and Th17-related cytokines (IFN-γ, IL-17, IL-2, and IL-6) increased, and the immunosuppressive function of Treg cells significantly decreased [24]; furthermore, the expression of the cytokine IL-10 also significantly decreased, suggesting that Tim-3 plays important roles in maintaining homeostasis in sepsis in both humans and a mouse model [22,23]. In addition to evidence from animal studies, in rheumatoid arthritis patients, higher Gal-9 and Tim-3 expression was found to be associated with better clinical outcomes and/or reduced disease severity. which provides additional evidence that Gal-9 exerts anti-inflammatory activity [25].

Tim-3 expression is dysregulated during the septic pathological process and is a negative immune regulator correlated with the severity of sepsis. The expression of Tim-3 in patients with severe sepsis was significantly lower than that in patients with mild sepsis [26]. Consistent with this finding, in a mouse model of sepsis, Tim-3 expression was significantly reduced in the acute/severe phase, and blockade of the Tim-3 pathway by Tim-3 antibodies and Tim-3 fusion proteins reduced the survival rate, increased proinflammatory factor release, and exacerbated septic injury [26]. In an *in vitro* study of sepsis, lipopolysaccharide (LPS) induced upregulation of inflammatory factors, such as IFN-γ, TNF-α, and IL-17, indicating excessive proliferation and activation of Th1 and Th17 cells [27], while Gal-9 inhibited the upregulation of these factors and promoted the production of the Th2 and Treg cell-related cytokines IL-4 and IL-10 [28].

These results indicate that the Gal-9/Tim-3 pathway can regulate Th17/Treg cells and related secretion factors in sepsis and kidney diseases. Our previous study found that IL-17 knockout improved SA-AKI and increased IL-10 expression while reducing IL-6 levels [29]. This finding indicates that IL-17 is involved in the pathogenesis of SA-AKI. In addition, in an IRI-AKI model, increasing the percentage of Treg cells and the secretion of IL-10 significantly improved AKI, while clearing Treg cells accelerated the deterioration in AKI and reduced IL-10 levels [30].

In our present study, we further confirmed that the number of IL-10-secreting Treg cells in SA-AKI was significantly reduced, while the number of IL-17-secreting Th17 cells was significantly increased. After exogenous Gal-9-mediated activation of the Gal-9/Tim-3 pathway, the number of IL-17-secreting Th17 cells decreased, and the number of IL-10-secreting Treg cells increased. In addition, blockade of the Gal-9/Tim-3 pathway with Tim-3 further aggravated the local Th17/Treg imbalance in the kidneys of septic mice, resulting in an increase in Th17 cells and a decrease in the number of IL-10-secreting Treg cells. In summary, Th17/Treg cells and IL-17/IL-10 secreted by Th17/Treg cells are important pathogenic factors involved in SA-AKI. Inducing Th17/Treg cells to establish a new balance may become a new target for SA-AKI treatment. The Gal-9/Tim-3 pathway may be an important mechanism through which the Th17/Treg ratio can be altered. Regulation of the Th17/Treg balance by manipulating the Gal-9/Tim-3 pathway may become a breakthrough treatment strategy.

The present study revealed that the administration of MSCs significantly reversed the Th1/Th2 balance in mice with SA-AKI, resulting in a significant decrease in the percentages of IL-17-secreting Th17 cells and a significant increase in IL-10-secreting Treg cells. In addition, activation of the Gal-9/Tim-3 pathway by exogenous Gal-9 reduced the number of IL-17-secreting Th17 cells and increased the number of IL-10-secreting Treg cells. Blocking the Gal-9/Tim-3 pathway with Tim-3 further aggravated the local Th17/Treg imbalance in the kidneys of septic mice, resulting in an increase in Th17 cells and a decrease in IL-10-secreting Treg cells. Previous studies have found that MSCs can induce IL-10 secretion by Treg cells in an IRI-AKI model [30]. Our previous studies demonstrated that MSCs inhibit the secretion of IL-17 by Th17 cells and increase IL-10 expression in mice with SA-AKI [3]. In addition, MSCs can inhibit T-cell function through the secretion of Gal-1 and Gal-3 [13]. Therefore, we speculate that reestablishing the Th17/Treg balance using MSCs that act through the Gal-9/Tim-3 pathway might be an important strategy to achieve protection against SA-AKI.

Our study has some limitations. For example, although our previous study verified that injected MSCs do not home to the kidney [3], we did not further verify how MSCs affect the Gal-9/Tim-3 pathway in the kidney. Therefore, in the future, using *in vitro* experiments, we will further verify whether MSCs affect the Gal-9/Tim-3 pathway through the secretion of Gal-9 or through other mechanisms, such as exosomes derived from MSCs or other paracrine mechanisms [31].

## Conclusions

In summary, our findings suggest that MSCs can ameliorate SA-AKI by altering the Th1/Th2 cell balance, and the important mechanism by which MSCs protect against SA-AKI may be mediated through the Gal-9/Tim-3 pathway. Our study offers additional evidence that administered MSCs protect against SA-AKI by remodeling immune homeostasis and sheds new light on the mechanisms underlying the beneficial effects of MSCs in AKI.
